# Association of SDF-1-3′ Gene A Variant with Diabetic Retinopathy in the Hungarian Population

**DOI:** 10.3390/ijms25158036

**Published:** 2024-07-23

**Authors:** Monika Ecsedy, Illes Kovacs, Andrea Szigeti, Hajnalka Horvath, Lilla Lenart, Zsuzsanna Recsan, Timea Medveczki, Zoltan Zsolt Nagy, Andrea Fekete

**Affiliations:** 1 Department of Ophthalmology, Semmelweis University Budapest, 1085 Budapest, Hungary; kovacs.illes@semmelweis.hu (I.K.); szigeti.andrea@semmelweis.hu (A.S.); hajnalkahorvath88@gmail.com (H.H.); recsan.zsuzsanna@semmelweis.hu (Z.R.); nagy.zoltan.zsolt@semmelweis.hu (Z.Z.N.); 2 Department of Clinical Ophthalmology, Faculty of Health Sciences, Semmelweis University Budapest, 1085 Budapest, Hungary; 3 Department of Ophthalmology, Weill Cornell Medical College, New York, NY 10065, USA; 4 MTA-SE Lendület Diabetes Research Group, Hungarian Academy of Sciences and Semmelweis University, 1085 Budapest, Hungary; lenart.lillaa@gmail.com (L.L.); medveczki.timea@phd.semmelweis.hu (T.M.); fekete.andrea1@semmelweis.hu (A.F.)

**Keywords:** stromal cell-derived factor 1, SDF-1-3′ (c801G > A) polymorphism, diabetic maculopathy, central retinal thickness

## Abstract

We investigated the association between the SDF-1-3′ (c801G > A) variant and the development of diabetic macular edema (DME) or proliferative diabetic retinopathy (PDR) in a Hungarian cohort. SDF-1-3′ (c801G > A) was genotyped in 103 patients with diabetic retinopathy and 31 age- and sex-matched non-diabetic controls. Central retinal and choroidal thickness was measured by swept-source optical coherence tomography. The distribution of heterozygous and homozygous SDF-1-3′ (c801G > A) genotypes was similar in diabetic and control subjects. The SDF-3′(c801AA) genotype was associated with DME (*n* = 94 eyes, allele distribution *p* = 0.006, genotype distribution *p* = 0.01 OR: 2.48, 95% CL: 1.21–5.08) in both univariable and multivariable modelling, independent of duration and type of diabetes, HbA1C, hypertension and microalbuminuria (*p* = 0.03). DME occurred earlier in patients carrying the SDF-1 (c801A) allele (Kaplan–Meier analysis, log-rank test *p* = 0.02). A marginally significant association was found between the presence of the SDF-1 (c801A) allele and the development of PDR (*n* = 89 eyes, *p* = 0.06). The SDF-1-3′ (c801A) allele also showed a correlation with central retinal (*p* = 0.006) and choroidal (*p* = 0.08) thickness. SDF-1-3′ (c801G > A) is involved in the development of macular complications in DM independent of critical clinical factors, suggesting that SDF-1 may be a future therapeutic target for high-risk patients, especially those carrying the SDF-1 (c801A) allele.

## 1. Introduction

Diabetic retinopathy (DR) is the leading cause of blindness among the working population in developed countries. Its prevalence is estimated to be 27.0%, which means there are 0.4 million cases of blindness worldwide [1]. Besides various metabolic, environmental, and other factors, genetic predisposition plays a significant role in the onset of DM-induced sight-threatening complications, primarily DM-associated retinopathy (PDR) and maculopathy (DME) [2,3].

Although considerable efforts have been made to understand the pathophysiology of these feared eye complications, the exact mechanism is still unknown. Since inflammation, angiogenesis, and angiostatic properties play a crucial role in PDR and DME, various cytokines and chemokines have already been studied for a long time [4,5].

Stromal cell-derived factor-1 (SDF-1) is an active chemokine that influences the trafficking and migration of endothelial progenitor cells [6,7]. Accumulating experimental and clinical data suggest that SDF-1 plays a significant role in the micro- and macrovascular complications of DM. In the recent CATIS trial, serum SDF-1 levels were independently associated with a higher cardiac autonomic neuropathy and cardiovascular risk score and recurrent strokes in diabetic patients, suggesting a causative interaction between plasma SDF-1 levels and the development of cardiovascular events, including stroke, and decreased all-cause mortality in diabetic patients [8,9]. Regarding ophthalmological complications, SDF-1 plays a crucial role in developing PDR via promoting VEGF-mediated neoangiogenesis [10,11,12], and it is also a key factor of DME by increasing microvascular permeability. Furthermore, vitreous SDF-1 is upregulated in diabetic patients with PDR and clinically significant macular oedema (CSME) [11,12,13,14].

SDF-1 has two transcriptional splice variants: sdf-1α and sdf-1β. The nucleotide transition—G to A—of the sdf-1-3′ is located at nucleotide position 801 in the 3′ untranslated region (3′UTR) of the sdf-1β transcript. SDF-1 (c801AA) homozygosity is associated with higher SDF-1 production [14]. Several data showing that the SDF-1-3′ (c801G > A) variant is associated with the early onset of T1DM in French, Japanese and Iranian populations [15,16] have already been reported; however, its relevance in diabetic retinopathy has only been investigated in one study (published in 2023). Peng et al. found that the SDF-1 (c801AA) genotype was ten times more common in Taiwanese patients with NPDR or PDR [17]. However, they did not analyze its association with DME.

This study investigates the SDF-1 (c801G > A) variant in diabetic patients in Hungary to assess its relevance in the development of DM-associated PDR, with a particular focus on DME. Furthermore, the association of the SDF-1 (c801G > A) variant was also evaluated after adjustment for known (metabolic or environmental) risk factors.

## 2. Results

### 2.1. Patient Characteristics

The baseline characteristics of the various diabetic and control groups are presented in Table 1.

### 2.2. Genotype Distribution and Allele Frequencies

Hardy Weinberg’s criteria were fulfilled in all groups. Table 2 summarizes the allele and genotype frequencies of SDF-1 (c801G > A) in the diabetic and control groups.

Table 3 summarizes the SDF-1 (c801G > A) variant genotypes and allele frequencies in different severity groups of diabetic retinopathy.

### 2.3. Genetic Association with Sight-Threatening Retinal Complications

The SDF-1 (c801G > A) variant was a significant predictor of DME in the multivariable regression model (OR: 2.48, 95% CL: 1.21–5.08; *p* = 0.01) after adjusting for the effect of risk factors for DME, such as the duration of DM, HbA1C level, hypertension, and microalbuminuria. In addition, the SDF-1 (c801G > A) variant did not predict PDR in our cohort.

### 2.4. Association of Macular and Choroidal Thickness with SDF1 (c801A) Allele

**Figure 1:**
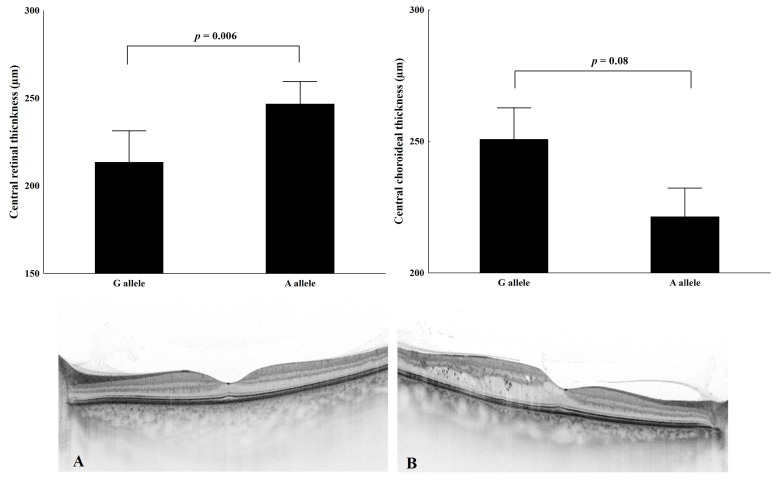
Association of SDF-1-3′A and G alleles with central retinal thickness and central choroidal thickness. Representative images of two patients of our study cohort: Image and layers of the retina and the choroid by swept-source optical coherence tomography (DRI OCT Triton, Topcon Co., Tokyo, Japan). (**Panel** (**A**)): A 55-year-old women with type-1 diabetes mellitus without any sign of diabetic retinopathy or diabetic macula oedema and standard choroidal thickness SDF-1 (c801GG) genotype. (**Panel** (**B**)): A64-year-old man with type-1 diabetes mellitus, slight diabetic macular oedema with thinner choroid; SDF-1 (c801AA) genotype. Note: *p*-values were calculated using the Kruskal–Wallis test.

As shown in Figure 1, the central macular thickness was significantly higher (*p* < 0.006), and central choroidal thickness was lower in DM patients with the presence of the SDF-1 (c801A) allele (*p* < 0.08).

**Figure 2:**
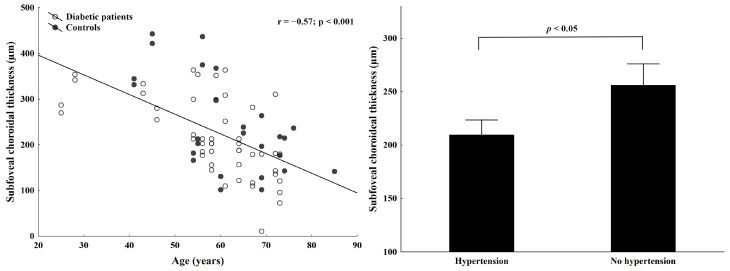
Regression analysis between age and subfoveal choroidal thickness in the diabetic and control study groups (**left**) and the effect of hypertension on choroidal thickness in the whole cohort (**right**).

As age-dependent thinning is a crucial factor in determining the role of the choroid in retinal pathology [18,19], we also performed regression analysis. We found a significant correlation between age and subfoveal choroidal thickness in diabetic and control subjects (Figure 2 left panel, *p* < 0.001, r = −0.57). Hypertension was also a significant predictor of central choroidal thinning in our DM patients (Figure 2 right panel, *p* < 0.05).

### 2.5. Onset of DME and Genetic Background

**Figure 3:**
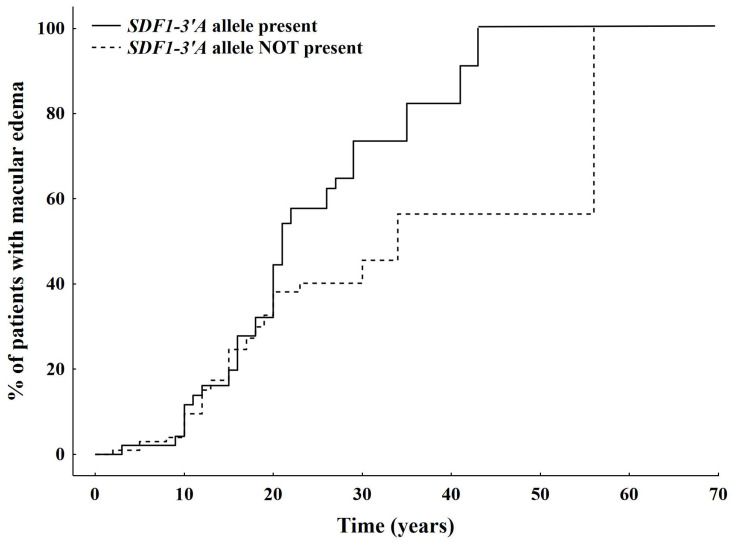
Comparison of time to DME in the eyes of diabetic patients with and without the presence of the SDF-1 (c801A) allele. Log-rank test for significance between groups *p* = 0.02.

Kaplan–Meier analysis showed that DME occurs significantly earlier in patients carrying the SDF-1 (c801A) allele (Figure 3, log-rank test, *p* = 0.02).

## 3. Discussion

DM, a metabolic disease, is influenced by a complex interplay of genetic and environmental factors. Importantly, we identified genetic factors as fundamental predictors critical in determining the diverse patterns of progression of diabetic retinopathy. In line with the Eye Diseases Prevalence Research Group findings, our research has classified diabetic retinopathy into primary outcomes: sight-threatening retinopathy, consisting of PDR, DME, or both [3].

This study confirmed that the SDF-1 (c801AA) genotype increases the risk of DME in diabetic patients. We also showed that the SDF-1 (c801AA) genotype was significantly associated with the development of DME, independent of the duration and type of DM or other factors such as HbA1C, hypertension, or microalbuminuria. Previous studies also concluded that these systemic markers are relevant factors in the development of diabetic retinopathy; however, these factors per se did not influence disease onset and the progression of the disease, presumably due to their large interindividual variability [2,3,20,21,22,23].

Since changes in retinal thickness are known biomarkers of DME progression and prognosis, we also investigated the association of the SDF-1 (c801A) allele with central macular retinal thickness. We showed that the central macular retinal thickness was increased in patients carrying the SDF-3′ (c801A) allele. In addition, DME developed earlier in carriers of the SDF-1 (c801A) allele. In other words, the proportion of patients living without DME for a given time was significantly lower in diabetic patients carrying the SDF-1 (c801A) allele [24].

The signaling pathway of SDF-1 and its receptor CXC chemokine 4 (CXCR4) has been implicated in angiogenesis, tumor growth, embryogenesis, and wound healing [25,26,27]. In the human retina, the localization of SDF-1 is mainly to the inner photoreceptor matrix and retinal pigment epithelial (RPE) cells, and the localization of CXCR4 is to the inner segment of the photoreceptors [28]. When SDF-1 binds to its receptor (CXCR4) on the endothelial cells, it induces angiogenesis by releasing vascular endothelial growth factors [29]. At the same time, VEGF has been reported to induce the expression of CXCR4 in endothelial cells [30]. In an in vitro study by Wu et al., the treatment of human retinal vascular endothelial cells (hRVECs) with high glucose increased the protein levels of SDF-1 and CXCR4, and the CXCR4 antagonist inhibited the expression levels of angiogenesis-related proteins in hRVECs [31].

Our study has led to a new hypothesis on the mechanism predisposing to the development of macular complications in patients carrying the SDF-1 (c801A) allele. Retinal endothelial cells and occluding gap junction proteins, responsible for tight junctions between endothelial cells, are critical in preventing retinal vascular leakage. Butler et al. showed that occludin expression by retinal endothelial cells is decreased with increasing SDF-1 levels [10,32]. The carrier status of SDF-1 (c801A) leads to increased vitreous expression and upregulated SDF-1 secretion. This increased chemokine pool triggers the homing of endothelial progenitor cells to sites of vascular leakage. Based on these previous data, we propose that this mechanism may be activated in our patients carrying the SDF-1 (c801A) allele, thereby predisposing them to the development of macular complications such as intraretinal fluid accumulation and increased macular thickness. This hypothesis opens up new perspectives for further research in this field.

In addition to retinal thickening, central choroidal thickness (CT) was thinner in our diabetic patients carrying the SDF-1 (c801A) allele; however, age and hypertension seemed to have a more significant predictive value for choroidal thinning. The importance of CT as a marker for diabetic retinopathy has been in the spotlight in recent years. However, the results are controversial. Regatieri et al. [33] reported no difference between non-proliferative retinopathy and healthy subjects, whereas CT was decreased in patients with PDR and DME. Similarly, in a multicenter study, Gerendas et al. [34] found a thinner choroid in DME patients than in healthy subjects. In contrast, Querques et al. [35] reported that although the diabetic group in their study had significantly thinner choroids than controls, there was no correlation with the severity of retinal pathology. Vujosevic et al. [36] found no difference between controls and diabetic patients, and DME did not affect choroidal thickness. At the same time, choroidal thickening has also been demonstrated in diabetic patients by Xu et al. and Yazici et al. [37,38] who found a correlation with diabetic neuropathy.

These controversial results may be due to several factors. First, most of these studies were retrospective and included patients treated with photocoagulation or anti-vascular endothelial growth factor (anti-VEGF) agents. In addition, most studies used spectral domain OCT, which only allows for the manual measurement of the choroid at specific points and is unsuitable for choroidal mapping. In our research, to increase the accuracy, we did not include healthy patients, only treatment-naive diabetic patients. We used a swept-source OCT device, allowing for a more detailed examination of the choroid and choroidal mapping.

We have previously published data on choroidal changes measured by SS-OCT. We found that in eyes with treatment-naive diabetic retinopathy, DM and the severity of diabetic retinopathy significantly influenced CT, even after adjusting for confounding systemic factors such as disease duration [39].

Regarding the molecular mechanism of choroidal changes in diabetes and its relationship to SDF-1, we only have in vitro studies. In postmortem eyes, the presence of SDF-1 and CXCR4 was most prominent in RPE cells and choroidal stroma by immunolocalization, and circulating cells, presumably leucocytes, were most intensely positive for CXCR4 [28]. Xu et al. [40] also used an animal model to investigate the expression of genes that may affect the blood–retinal barrier, including the cytokine IL-1β, the chemokines CCL2 and SDF-1, and the growth factors FGF and VEGF, in the retina and the RPE/choroid of mice undergoing extracapsular lens extraction. They found that the expression of these genes was significantly upregulated in the retina and the choroid. However, the exact pathogenesis of choroidal thinning and the role of the SDF-1 and CXCR4 signaling pathways in diabetic eyes remain unknown.

Although here we also found a strong correlation between DME and the SDF-1 (c801A) allele, there was no association with the development of PDR. As this correlation has already been published in T2DM and NPDR patients in a recent Taiwanese study [24], we assume that the relatively small sample size or the inclusion of T1DM patients may explain the lack of significance and the discrepancy with the literature.

Our study has limitations, such as the small sample size and the inclusion of both T1DM and T2DM. It should also be considered that our study population was white Caucasians of Hungarian ethnic origin. Therefore, the possibility of ethnicity as a confounding factor cannot be excluded.

In conclusion, our data suggest that the SDF-1 (c801G > A) variant is a relevant contributor to the development of DME. The binary logistic regression analysis, including the most studied influencing clinical parameters, suggested that this polymorphism remains a strong, independent, and predictive factor for the development of the disease. Finally, OCT measurements support this association between genetic variants and macular pathology.

In this direction of incorporating genetic findings into DME clinical practice, SDF-1-3′ (c801G > A) polymorphism may be an ideal biomarker and also a future therapeutic target for high-risk diabetic retinopathy patients, especially those carrying the SDF-1 (c801A) allele. Regarding possible interventions targeting the SDF-di1 pathway, among the existing therapies, intravitreal injection of triamcinolone reduced vitreous SDF-1 levels in patients with DR [10]. Furthermore, as was published in the CATIS study, elevated plasma SDF-1 was significantly associated with an increased risk of recurrent stroke and cardiovascular events at one year only in ischaemic stroke patients with diabetes mellitus but not in those without diabetes mellitus [8]. SDF-1 antagonists may be helpful in the future, not only for ocular complications but also for the prevention of recurrent stroke, cardiovascular events, and all-cause mortality.

However, larger cohorts need to be studied to assess the potential benefit of SDF-1 (c801G > A) genotyping.

## 4. Materials and Methods

### 4.1. Study Population

One hundred and three Hungarian patients with DM and thirty-one non-diabetic patients were recruited from the Department of Ophthalmology outpatient clinic of Semmelweis University, Budapest, Hungary. The study was conducted in accordance with the tenets of the Declaration of Helsinki and the applicable national and local ethics committee and institutional review board requirements. Ethical approval was obtained from the Institutional Review Board (Semmelweis University Regional and Institutional Committee of Sciences and Research Ethics, TUKEB TUKEB 839/PI/010). Written informed consent was obtained from each patient before the study.

Inclusion criteria: All patients with any signs of diabetic retinal microvascular complications were included in the study.

Exclusion criteria: Patients with a history of previous intraocular surgery, ocular trauma, or any other retinal or neurological disease (e.g., multiple sclerosis), intraocular inflammation, or tumor were excluded from the study. Patients with prior anti-VEGF or laser treatment for DME or PDR were also excluded from the study because of the modifying effect of these treatment modalities on retinal and choroidal thickness values.

The control group consisted of randomly selected sex- and age-matched unrelated volunteers who were referred for spectacle prescription or general routine eye care.

All cases and controls were Caucasian and Hungarian. The inclusion and exclusion criteria were the same for the DM and age -and sex-matched control groups, except that patients in the control group had no evidence of DR.

### 4.2. Clinical History and Routine Laboratory Parameters

Clinical data, anthropometric data, patient and family history, and duration of DM were recorded. The diagnosis of T1DM and T2DM was based on the American Diabetes Association criteria [24]. Routine biochemical parameters (fasting plasma glucose, haemoglobin A1C, microalbuminuria) were measured at baseline. Antihypertensive treatment was also recorded, and a patient was considered to have hypertension if they were treated with any antihypertensive medication.

### 4.3. Ophthalmological Examination

The best corrected visual acuity was measured using a Snellen chart. Retinal microvascular features were assessed by fundus biomicroscopy. Diagnostic criteria for diabetic retinopathy were applied according to the 2008 consensus guidelines of the Hungarian Diabetes Association and the Society of Hungarian Ophthalmologists based on data from the American Academy of Ophthalmology [24,41,42].

DME was determined by any retinal thickening or hard exudates in the posterior pole on biomicroscopic examination (defined as clinically significant macular edema essential contribution of the ETDRS clinical trials) [24,41,42]. At the same time, to obtain objectively measurable data and to detect less-prominent intraretinal macular pathology, central retinal thickness and central choroidal thickness were measured in all participants using swept-source optical coherence tomography (DRI OCT Triton, Topcon Co., Tokyo, Japan) using the 3D Macular Volumetric Raster Scan Protocol. The acquired scans were analyzed using Triton software version 3.

PDR was identified (according to the ETDRS severity scale) if there was evidence of neovascularization during the fundus biomicroscopy, with or without vitreous haemorrhage. Fluorescein angiography was also performed in ambiguous cases to locate the leaky neovascular structures [43,44].

### 4.4. DNA Extraction and Genotyping

Blood samples remaining after the routine examination were used for genotyping. Genomic DNA was extracted from whole blood using the QIAamp Blood Mini kit (Qiagen, Hilden, Germany). Genotyping of the SDF-1 (c801G > A) variant was performed as follows: DNA fragments were amplified in a reaction mix containing 10% buffer, 2 MgCl_2_, dNTP, sdf-1 specific primers (F: CAGTCAACCTGGGCAAAGCC, R: CCTGAGAGTCCTTTTGCGGG) and recombinant Taq polymerase (Invitrogen, Budapest, Hungary), using the following PCR conditions: initial denaturation at 94 °C for 5 min, followed by 35 cycles of denaturation at 94 °C for 1 min, annealing at 55 °C for 1 min and extension at 72 °C for 1 min, ending with a final extension at 72 °C for 5 min and cooling to 4 °C in a Perkin-Elmer Thermo Cycler (PE, Model 2400; Norwalk, CT, USA). The PCR products were digested with Msp1 (Sigma Chemical Co., Budapest, Hungary) at 37 °C for 4 h. The cleavage products were electrophoresed on a 3% agarose gel and stained with GelRed™ (Biotium, CA, USA).

### 4.5. Statistical Analysis

Statistical analysis was performed using SPSS 21.0 (IBM Inc., Chicago, IL, USA). The Shapiro–Wilk test was used for normal distribution, and parametric or non-parametric statistical tests were used to compare data from the two study groups. We calculated allele and genotype frequencies in patients and healthy controls through direct counting.

The Hardy–Weinberg equilibrium was assessed for both variants in diabetic and control subjects by comparing the observed and expected frequencies of the genotypes using chi-squared analysis. To determine the effect of the SDF-1 (c801G > A) variant among multiple predictors on the development of DME or PDR, multivariable regression analyses were performed using binomial logistic regression models, adding duration and type of DM, hypertension, microalbuminuria and HBA1C level as covariates to adjust for their effect on the development of DME or PDR. Kaplan–Meier life table analysis was used to construct curves for the time to onset of diabetic maculopathy, and the log-rank test was used to compare their distribution. The significance level was set at *p* < 0.05 for all statistical analyses.

## Figures and Tables

**Table 1:** Laboratory parameters and retinal and choroidal thickness values in the central macular area of the control and the diabetic groups, *p* < 0.05 T1DM vs. T2DM; diabetic vs. control groups, respectively. Note: The Student *t*-test is for continuous variables, and the Chi-square test is for categorical variables. PDR: proliferative diabetic retinopathy, DME: diabetic macular oedema, DM: diabetes mellitus, T1DM: type 1 diabetes mellitus, T2DM: type 2 diabetes mellitus, NA: not applicable.

Variables	Controls (*n* = 31)	T1DM (*n* = 28)	T2DM (*n* = 75)	*p*-Value (T1 vs. T2DM2)	*p*-Value (DM vs. Control)
Age (years)	61.0 ± 15.0	52.7 ± 18.0	64.6 ± 9.76	0.001	0.63
Gender (male/female)	13/24	13/15	37/38	0.698	0.06
PDR (eyes)	NA	19/28	40/75	0.015	NA
DME (eyes)	NA	14/28	30/75	0.295	NA
DM duration (years)	NA	25.3 ± 12.3	16.6 ± 8.12	0.011	NA
HbA1C (mmol/L)	<6.5	8.07 ± 1.51	7.59 ± 1.73	0.307	NA
Microalbuminuria (Y/N)	0/31	13/28	12/75	0.002	NA
Hypertension (patients n)	11/31	20/28	75/75	0.007	<0.001
CRT (microns, eyes)	270 ± 49.8	277 ± 95.9	362 ± 188	0.006	0.02
CSI (microns, eyes)	249 ± 109	245 ± 67.2	225 ± 80.9	0.23	0.09

**Table 2:** SDF-1 (c801G > A) allele and genotype frequencies in control and diabetic (DM) patients, *p* < 0.05; DM vs. control groups, resp. Note: *p*-values were calculated using the Chi-squared test, DM: diabetes mellitus.

Alleles	A Allele (*n*)	G Allele (*n*)	*p*-Value
Control patients (*n* = 62)	14 (22.5%)	48 (77.5%)	0.48
DM patients (*n* = 206)	58 (27.2%)	148 (71.8%)	
**Genotypes**	**AA**	**AG + GG**	
Control patients (*n* = 31)	3 (9.7%)	28 (90.3%)	0.99
DM patients (*n* = 103)	10 (9.7%)	93 (90.3%)	
**Genotypes**	**AA + AG**	**GG**	
Control patients (*n* = 31)	11 (35.5%)	20 (64.5%)	0.38
DM patients (*n* = 103)	48 (46.6%)	55 (54.4%)	

**Table 3:** SDF-1 (c801G > A) allele and genotype frequencies in the eyes of diabetic patients with/without DME or with/without PDR. *p* < 0.05 between groups of eyes with/without DME or with/without PDR of DM patients; resp. Note: *p*-values were calculated using the Chi-squared test, PDR: proliferative diabetic retinopathy, DME: diabetic macular edema, DM: diabetes mellitus.

Alleles	An Allele (*n*)	G Allele (*n*)	*p*-Value
DM eyes without PDR (*n* = 234)	73 (31.2%)	161 (68.8%)	0.14
DM eyes with PDR (*n* = 178)	43 (24.2%)	135 (75.8%)	
DM eyes without DME (*n* = 224)	50 (22.3%)	174 (77.7%)	0.006
DM eyes with DME (*n* = 188)	66 (35.1%)	122 (64.9%)	
**Genotypes**	**AA + AG**	**GG**	
DM eyes without PDR (*n* = 117)	60 (51.3%)	57 (48.7%)	0.16
DM eyes with PDR (*n* = 89)	36 (40.4%)	53 (59.5%)	
DM eyes without DME (*n* = 112)	43 (38.4%)	69 (61.6%)	0.01
DM eyes with DME (*n* = 94)	53 (56.38%)	41 (43.61%)	

## Data Availability

Data are contained within the article.

## References

[1] Thomas R., Halim S., Gurudas S., Sivaprasad S., Owens D. (2019). IDF Diabetes Atlas: A review of studies utilising retinal photography on the global prevalence of diabetes related retinopathy between 2015 and 2018. Diabetes Res. Clin. Pract..

[2] Cabrera A.P., Monickaraj F., Rangasamy S., Hobbs S., McGuire P., Das A. (2020). Do genomic factors play a role in diabetic retinopathy?. J. Clin. Med..

[3] Warpeha K.M., Chakravarthy U. (2003). Molecular genetics of microvascular disease in diabetic retinopathy. Eye.

[4] Aldhahi W., Hamdy O. (2003). Adipokines, inflammation, and the endothelium in diabetes. Curr. Diabetes Rep..

[5] Luster D.A. (1998). Chemokines—Chemotactic cytokines that mediate inflammation. N. Engl. J. Med..

[6] Aiuti A., Webb I.J., Bleul C., Springer T., Gutierrez-Ramos J.C. (1997). The chemokine SDF-1 is a chemoattractant for human CD34^+^ hematopoietic progenitor cells and provides a new mechanism to explain the mobilization of CD34^+^ progenitors to peripheral blood. J. Exp. Med..

[7] Bleul C., Fuhlbrigge R.C., Casasnovas J.M., Aiuti A., Springer T. (1996). A Highly Efficacious Lymphocyte Chemoattractant, Stromal Cell-derived Factor-1 (SDF-1). J. Exp. Med..

[8] You S., Chen H., Miao M., Du J., Che B., Xu T., Liu C.-F., Zhang Y., He J., Zhong X. (2023). Prognostic significance of plasma SDF-1 in acute ischemic stroke patients with diabetes mellitus: The CATIS trial. Cardiovasc. Diabetol..

[9] Liu W.-S., Hua L.-Y., Zhu S.-X., Xu F., Wang X.-Q., Lu C.-F., Su J.-B., Qi F. (2022). Association of serum stromal cell-derived factor 1 levels with EZSCAN score and its derived indicators in patients with type 2 diabetes. Endocr. Connect..

[10] Butler J.M., Guthrie S.M., Koc M., Afzal A., Caballero S., Brooks H.L., Mames R.N., Segal M.S., Grant M.B., Scott E.W. (2005). SDF-1 is both necessary and sufficient to promote proliferative retinopathy. J. Clin. Investig..

[11] Humpert P.M., Neuwirth R., Battista M.J., Voronko O., von Eynatten M., Konrade I., Rudofsky G., Wendt T., Hamann A., Morcos M. (2005). SDF-1 Genotype Influences Insulin-Dependent Mobilization of Adult Progenitor Cells in Type 2 Diabetes. Diabetes Care.

[12] Sei S., O’Neill D.P., Stewart S.K., Yang Q., Kumagai M., Boler A.M., Adde M.A., Zwerski S.L., Wood L.V., Venzon D.J. (2001). Increased level of stromal cell-derived factor-1 mRNA in peripheral blood mononuclear cells from children with AIDS-related lymphoma. Cancer Res..

[13] Brooks H.L., Caballero S., Newell C.K., Steinmetz R.L., Watson D., Segal M.S., Harrison J.K., Scott E.W., Grant M.B. (2004). Vitreous Levels of Vascular Endothelial Growth Factor and Stromal-Derived Factor 1 in Patients with Diabetic Retinopathy and Cystoid Macular Injection of Triamcinolone. Arch. Ophthalmol..

[14] Winkler C., Modi W., Smith M.W., Nelson G.W., Wu X., Carrington M., Dean M., Honjo T., Tashiro K., Yabe D. (1998). Genetic restriction of AIDS pathogenesis by an SDF-1 chemokine gene variant. Science.

[15] Ide A., Kawasaki E., Abiru N., Sun F., Fukushima T., Takahashi R., Kuwahara H., Fujita N., Kita A., Oshima K. (2003). Stromal-cell derived factor-1 chemokine gene variant is associated with type 1 diabetes age at onset in Japanese population. Hum. Immunol..

[16] Dubois-Laforgue D., Hendel H., Caillat-Zucman S., Zagury J.-F., Winkler C., Boitard C., Timsit J. (2001). A common stromal cell–derived factor-1 chemokine gene variant is associated with the early onset of type 1 diabetes. Diabetes.

[17] Peng S.-Y., Chuang C.-C., Hwang Y.-S., Yen C.-H., Lee C.-Y., Yang S.-F. (2023). Association of SDF-1 and its receptor CXCR4 polymorphisms on the susceptibility of diabetic retinopathy in the Taiwanese population. Front. Genet..

[18] Ikuno Y., Kawaguchi K., Nouchi T., Yasuno Y. (2010). Choroidal thickness in healthy Japanese subjects. Investig. Opthalmol. Vis. Sci..

[19] Ouyang Y., Heussen F.M., Mokwa N., Walsh A.C., Durbin M.K., Keane P.A., Sanchez P.J., Ruiz-Garcia H., Sadda S.R. (2011). Spatial distribution of posterior pole choroidal thickness by spectral domain optical coherence tomography. Investig. Opthalmol. Vis. Sci..

[20] Ruiz-Medrano J., Flores-Moreno I., Peña-García P., Montero J.A., Duker J.S., Ruiz-Moreno J.M. (2014). Macular choroidal thickness profile in a healthy population measured by swept-source optical coherence tomography. Investig. Opthalmol. Vis. Sci..

[21] Kempen J.H., O’Colmain B.J., Leske M.C., Haffner S.M., Klein R., Moss S.E., Taylor H.R., Hamman R.F. (2004). The prevalence of diabetic retinopathy among adults in the United States. Arch. Ophthalmol..

[22] Hove M.N., Kristensen J.K., Lauritzen T., Bek T. (2006). The relationships between risk factors and the distribution of retinopathy lesions in type 2 diabetes. Acta Ophthalmol. Scand..

[23] Cunha-Vaz J., Ribeiro L., Lobo C. (2014). Phenotypes and biomarkers of diabetic retinopathy. Prog. Retin. Eye Res..

[24] American Diabetes Association (2002). The expert committee on the diagnosis and classification of diabetes mellitus* report of the expert committee on the diagnosis and classification of diabetes mellitus. Diabetes Care.

[25] Guleng B., Tateishi K., Ohta M., Kanai F., Jazag A., Ijichi H., Tanaka Y., Washida M., Morikane K., Fukushima Y. (2005). Blockade of the stromal cell–derived factor-1/CXCR4 axis attenuates in vivo tumor growth by inhibiting angiogenesis in a vascular endothelial growth factor–independent manner. Cancer Res..

[26] Dar A., Kollet O., Lapidot T. (2006). Mutual, reciprocal SDF-1/CXCR4 interactions between hematopoietic and bone marrow stromal cells regulate human stem cell migration and development in NOD/SCID chimeric mice. Exp. Hematol..

[27] Jin D.K., Shido K., Kopp H.G., Petit I., Shmelkov S.V., Young L.M., Hooper A.T., Amano H., Avecilla S.T., Heissig B. (2006). Cytokine-mediated deployment of SDF-1 induces revascularization through recruitment of CXCR4+ hemangiocytes. Nat. Med..

[28] A Bhutto I., McLeod D.S., Merges C., Hasegawa T., A Lutty G. (2006). Localisation of SDF-1 and its receptor CXCR4 in retina and choroid of aged human eyes and in eyes with age related macular degeneration. Br. J. Ophthalmol..

[29] Mirshahi F., Pourtau J., Li H., Muraine M., Trochon V., Legrand E., Vannier J.-P., Soria J., Vasse M., Soria C. (2000). SDF-1 activity on microvascular endothelial cells. Thromb. Res..

[30] Salcedo R., Wasserman K., Young H.A., Grimm M.C., Howard O.M.Z., Anver M.R., Kleinman H.K., Murphy W.J., Oppenheim J.J. (1999). Vascular endothelial growth factor and basic fibroblast growth factor induce expression of CXCR4 on human endothelial cells. Am. J. Pathol..

[31] Wu D., Jin L., Xu H. (2019). The effects of the CXCR4 antagonist, AMD3465, on Human Retinal Vascular Endothelial Cells (hRVECs) in a high glucose model of diabetic retinopathy. Med. Sci. Monit..

[32] Butler J.M. (2006). Role of Stromal Cell-Derived Factor 1 in Proliferative Retinopathy. Ph.D. Thesis.

[33] Regatieri C.V., Branchini L., Carmody J., Fujimoto J.G., Duker J.S. (2012). Choroidal thickness in patients with diabetic retinopathy analyzed by spectral-domain optical coherence tomography. Retina.

[34] Gerendas B.S., Waldstein S.M., Deak G., Hajnajeeb B., Zhang L., Bogunovic H., Abramoff M.D., Kundi M., Sonka M., Schmidt-Erfurth U. (2014). Three-dimensional automated choroidal volume assessment on standard spectral-domain optical coherence tomography and correlation with the level of diabetic macular edema. Am. J. Ophthalmol..

[35] Querques G., Lattanzio R., Querques L., Del Turco C., Forte R., Pierro L., Souied E.H., Bandello F. (2012). Enhanced depth imaging optical coherence tomography in type 2 diabetes. Investig. Opthalmology Vis. Sci..

[36] Akkaya S. (2018). Macular and peripapillary choroidal thickness in patients with keratoconus. Ophthalmic Surg. Lasers Imaging Retin..

[37] Xu J., Xu L., Du K.F., Shao L., Chen C.X., Zhou J.Q., Wang Y.X., You Q.S., Jonas J.B., Bin Wei W. (2013). Subfoveal choroidal thickness in diabetes and diabetic retinopathy. Ophthalmology.

[38] Yazici A., Sari E.S., Koc R., Sahin G., Kurt H., Ozdal P.C., Ermis S.S. (2016). Alterations of choroidal thickness with diabetic neuropathy. Investig. Opthalmology Vis. Sci..

[39] Horváth H., Kovács I., Sándor G.L., Czakó C., Mallár K., Récsán Z., Somogyi A., Nagy Z.Z., Ecsedy M. (2018). Choroidal thickness changes in non-treated eyes of patients with diabetes: Swept-source optical coherence tomography study. Acta Diabetol..

[40] Xu H., Chen M., Forrester J.V., Lois N. (2011). Cataract surgery induces retinal pro-inflammatory gene expression and protein secretion. Investig. Ophthalmol. Vis. Sci..

[41] Szemészeti K.A. (2005). Kollégium, A szemészeti szövõdmények terápiája diabetes mellitusban. Diabetologia Hungarica.

[42] Cheung N., Mitchell P., Wong T.Y. (2010). Diabetic retinopathy. Lancet.

[43] Conrath J., Giorgi R., Raccah D., Ridings B. (2004). Foveal avascular zone in diabetic retinopathy: Quantitative vs qualitative assessment. Eye.

[44] (1991). Early Treatment Diabetic Retinopathy Study Research Group. Classification of Diabetic Retinopathy from Fluorescein Angiograms. Ophthalmology.

